# Understanding supported self‐management for people living with a lower‐grade glioma: Implementation considerations through the lens of normalisation process theory

**DOI:** 10.1111/hex.14073

**Published:** 2024-05-11

**Authors:** Ben Rimmer, Tracy Finch, Michelle Balla, Lizzie Dutton, Sophie Williams, Joanne Lewis, Pamela Gallagher, Richéal Burns, Vera Araújo‐Soares, Fiona Menger, Linda Sharp, Ways Ahead study team

**Affiliations:** ^1^ Population Health Sciences Institute, Newcastle University Centre for Cancer Newcastle University Newcastle upon Tyne UK; ^2^ Department of Nursing, Midwifery and Health Northumbria University Newcastle upon Tyne UK; ^3^ Faculty of Medical Sciences Newcastle University Newcastle upon Tyne UK; ^4^ Newcastle upon Tyne Hospitals NHS Foundation Trust Newcastle upon Tyne UK; ^5^ School of Psychology Dublin City University Dublin Ireland; ^6^ Faculty of Science Atlantic Technological University Sligo Ireland; ^7^ Health and Biomedical Strategic Research Centre Atlantic Technological University Sligo Ireland; ^8^ Department for Prevention of Cardiovascular and Metabolic Disease, Centre for Preventive Medicine and Digital Health, Medical Faculty Mannheim Heidelberg University Heidelberg Germany; ^9^ School of Education, Communication and Language Sciences Newcastle University Newcastle upon Tyne UK

**Keywords:** healthcare professionals, lower‐grade gliomas, normalisation process theory, self‐management support

## Abstract

**Background:**

Supported self‐management can improve clinical and psychosocial outcomes in people with cancer; the considerations required to implement self‐management support (SMS) for people living with a lower‐grade glioma (LGG)—who often have complex support needs—are not known. We aimed to identify and understand these implementation considerations through the lens of normalisation process theory (NPT), from the perspectives of healthcare professionals (HCP) and people with LGG.

**Methods:**

We conducted semistructured interviews with HCPs who support adults with brain tumours (*n* = 25; 12 different healthcare professions), and people with LGG who had completed primary treatment (*n* = 28; male *n* = 16, mean age 54.6 years, mean time since diagnosis 8.7 years), from across the United Kingdom. Interviews were transcribed and inductive open coding conducted, before deductively mapping to constructs of NPT. We first mapped HCP data, then integrated data from people with LGG to explore alignment in experiences and perspectives.

**Results:**

We generated supporting evidence for all four NPT constructs and related subconstructs, namely: ‘Coherence’, ‘Cognitive participation’, ‘Collective action’ and ‘Reflexive monitoring’. Data from HCPs and people with LGG clearly demonstrated that effective SMS constitutes a collective activity. Key implementation considerations included: ensuring awareness of, and access to, support; building strong HCP‐support recipient relationships; and careful inclusion of close family and friends. We identified pertinent challenges, such as identifying support needs (influenced by the extent to which those with LGG engage in help‐seeking), resistance to support (e.g., technology literacy), training for HCPs and HCP cooperation.

**Conclusions:**

This study demonstrates the collective nature of, and provides insight into the individual roles within, supported self‐management. We outline considerations to operationalise, sustain and appraise the implementation of SMS for people with LGG.

**Patient or Public Contribution:**

People with brain tumours, and informal caregivers, were involved in the development of information materials and topic guides to ensure accessibility and pertinence. They also had opportunities to comment on interview findings.

## 1 INTRODUCTION

With rising survival rates and growing numbers of people living with and beyond the disease, cancer is increasingly considered a chronic condition. Self‐management in the context of cancer is defined as the ‘awareness and active participation by the person in their recovery, recuperation, and rehabilitation to minimise the consequences of treatment, and promote survival, health, and wellbeing’.^1^ There is an expanding evidence base to support the potential effectiveness of self‐management interventions for improving clinical, psychosocial, and economic outcomes in people with cancer.^2^, ^3^ However, self‐management is not one individual's responsibility; healthcare professionals (HCPs), family and friends have a crucial role in ensuring the person can effectively engage in self‐management.^4^ Indeed, self‐management strategies are more likely to be effective when planned together with support from HCPs.^5^

In 2020, there were an estimated 300,000 new diagnoses, worldwide, of primary brain and central nervous system tumours^6^; amongst the most common of these were gliomas, which can be high‐ or low‐grade.^7^ People with lower‐grade gliomas (LGG) have a life expectancy of 5–15 years following diagnosis,^8^, ^9^ and can experience wide‐ranging symptoms and impairments (e.g., fatigue, seizures, cognitive deficits) that adversely affect health‐related quality of life.^10^, ^11^ These impacts may persist long‐term, particularly concerning fatigue and emotional impact.^12^ Consequently, people with LGG may have prolonged, multifaceted supportive care needs; it is, therefore, important to identify how they can be supported and empowered to self‐manage their condition.

People with LGG have shown a willingness to engage in self‐management, reporting the use of a diverse and extensive number of self‐management strategies; the most common strategy type was ‘using support’ (e.g., receiving support from family).^13^ This complements the finding that people with brain tumours desire timely access to information and support from HCPs to help them self‐manage (e.g., development of shared self‐management care plans for support recipients and their family).^14^ However, little is known about how HCPs perceive their role in providing self‐management support (SMS) for people with LGG. In a study of advanced cancer (which did not focus on brain tumours), HCPs differed in their practices, adopting varied instructive, collaborative or advisory approaches to SMS.^15^

There was a recent ‘call to action’ for self‐management in cancer care, calling for a shift in care culture from people being passive recipients to active partners in their care, to embed co‐created person‐centred SMS.^16^ Therefore, attitudes towards, and the acceptability of SMS, are crucial implementation considerations^17^ which need to be understood from both the HCP and support recipient perspective. Furthermore, for SMS to be successful, the barriers to implementation at the organisational and HCP level also need to be understood and overcome.^18^

In other cancers, the few available studies indicate that key HCP barriers to implementation of SMS included time, communication between HCPs and appropriate knowledge and training^19^, ^20^; in addition, lack of HCP confidence in providing SMS led to reduced motivation.^21^ A competency framework has been developed to inform SMS training for cancer nurses^22^; however, healthcare organisations need to be ready and willing to implement SMS, which requires a process of change.^23^ For people with LGG specifically, the considerations required to implement SMS are poorly understood.

Normalisation process theory (NPT)^24^ offers a generalisable framework outlining the generative mechanisms of social action and the considerations required to implement a new practice into routine care. NPT has been used in a diverse range of healthcare settings to explain the implementation processes of complex interventions.^25^ Therefore, our study aimed, for the first time, to use the lens of NPT to identify and understand the considerations required to implement SMS for people living with an LGG, from the perspectives of HCPs and people with LGG.

## 2 METHODS

### 2.1 Design

This qualitative study, part of the wider Ways Ahead project,^26^ generated data on HCPs' and people with LGGs' experiences of (supporting someone) living with an LGG; the present analysis focused on the considerations that may influence the implementation of SMS. Ways Ahead was reviewed and approved by the Wales Research Ethics Committee (REC ref: 20/WA/0118).

### 2.2 Participants and recruitment

HCPs were eligible if they were a member of a relevant multidisciplinary team (MDT), involved in the care of adults with brain tumours (e.g., clinical nurse specialist); or were involved in the support of adults with brain tumours outside of National Health Service (NHS) care pathways (e.g., counsellors).

People with a diagnosis of grade 2 or 3 oligodendroglioma or grade 2 astrocytoma, based on histology or molecular features^27^ were eligible if they were aged ≥18 years at diagnosis, resident in the United Kingdom, and were stable under observation, or had completed primary treatment; hereafter, we refer to these as people with LGG.

We identified potentially eligible HCPs and people with LGG through collaborating NHS sites and The Brain Tumour Charity networks. People with LGG require multidisciplinary management, so in addition to consultant clinical oncologists and neurosurgeons, there are clinical nurse specialists, and some services may have additional roles (e.g., Occupational Therapist, Clinical Neuropsychologist). Therefore, we used purposive sampling to ensure HCP recruitment comprised a range of healthcare professions, across the United Kingdom. Recruitment of people with LGG comprised of a range of ages, sex, diagnoses and time since diagnosis (1–5, 6–10, >10 years).

For NHS sites, HCPs within their respective MDTs, and people with LGG identified from medical records, were given an information sheet by the principal investigator or another HCP at the site. For The Brain Tumour Charity networks, B. R. linked the information sheet to a study advertisement, which was disseminated through the charity's newsletters. HCPs and people with LGG were asked to register their interest by calling or emailing the study team; B. R. called each interested person to answer any potential questions, then if confirmed as eligible and willing to participate, arranged a convenient date and time for interview. We conducted recruitment between August 2020 and May 2022; recruitment continued until we judged that reasonable data sufficiency was achieved.^28^

### 2.3 Data generation

B. R. and L. D., both trained and experienced in qualitative research, remotely conducted semistructured interviews, via a phone or video call (e.g., Zoom or Teams), as per interviewee preference. Immediately before each interview, audio‐recorded consent was acquired, and demographic information was collected (e.g., from people with LGG: sex, age, diagnosis, treatment; from HCPs: profession, years working with people with brain tumours).

We used separate topic guides for HCP and people with LGG interviews (Files S1 and S2); each comprised open questions informed by the literature and expert knowledge. Both topic guides were reviewed by HCPs (J. L. and S. W.); the topic guide for people with LGG was also reviewed by a brain tumour Patient and Public Involvement panel and modified appropriately. Any new issues raised in an interview were added to the respective guide, to be explored in subsequent interviews.

For HCP interviews, participants were first asked to broadly reflect on their role in supporting people living with a brain tumour. We then explored participants' views on the support needs of people with LGG, how these needs are identified, what support is available following treatment completion, including their perception of, and role in supporting, self‐management and any challenges faced in providing support.

For interviews with people with LGG, participants were first asked to broadly reflect on life following their diagnosis. We then explored participants' views on how they had been impacted by the tumour and its treatment, how they had managed, their perceived support needs and whether support was received, including their experiences with seeking, receiving, and engaging with, healthcare support. People with LGG were offered a £20 voucher as a thank you for their time and given details of relevant charities and helplines on a postinterview sheet, which they could consult if they wanted further information or support.

Across both interview sets, we used probing questions to explore further, and all participants were afforded the opportunity to raise any additional issues of importance to them. We audio‐recorded each interview; interviews lasted on average 72 min (48–93 min) for HCPs and 102 min (54–167 min) for people with LGG.

### 2.4 Data analysis

Interviews were transcribed verbatim and anonymised; transcripts were checked for accuracy against the audio‐recordings. The present analysis aimed to identify and understand what might influence the implementation of SMS for people with LGG. We commenced analysis with an inductive, open coding approach; B. R. and M. B., both trained in qualitative analysis, independently generated initial codes following familiarisation with a subset of transcripts of people with LGG (*n* = 6 of 28) and HCPs (*n* = 5 of 25). The coding frame was refined following discussion between the researchers; the remaining transcripts were then coded by B. R. As coding progressed, findings and uncertainties were discussed with M. B. and L. S. Data sufficiency was reached when we judged that sufficient data had been generated to support and understand the implementation considerations for SMS.^28^

Following this, we deductively mapped our codes to the four constructs of NPT,^24^ namely: ‘Coherence’, ‘Cognitive participation’, ‘Collective action’ and ‘Reflexive monitoring’. Each construct has four related subconstructs that were used to guide the deductive mapping (e.g., ‘Cognitive participation’ encompasses *initiation*, *enrolment*, *legitimation*, and *activation*); construct descriptions are provided in Table 1. Initial deductive coding included discussion with L. S. and T. F., who is an expert in NPT; the mapping of codes was then revised and finalised. Overall, we examined how our codes corresponded to each generative mechanism of social action in the context of implementing SMS for people with LGG.

**Table 1:** Normalisation process theory construct definitions, key findings and supporting quotes.

NPT construct	Key finding	Supporting quotes
*Coherence—The sense‐making work that people do individually and collectively when they are faced with the problem of operationalising some set of practices*		
*Differentiation* Understanding how a set of practices are different from each other.	HCP: aim to promote independence BUT people with LGG: loss of independence	We want to make these patients as independent as we absolutely can. You know, we want them to take responsibility of their cancer, of how things are. HCP14 (Clinical Nurse Specialist) It's all about self‐management as well, trying to give them the strength and the confidence to access what they need when they need it and be a bit more independent. HCP33 (Clinical Nurse Specialist) I try and work around what the issue is so that the person can still stay independent in making themselves a meal, but they would do it differently. They just wouldn't do it from scratch in maybe the traditional way that they would do it. HCP49 (Occupational Therapist) The loss of my licence and independence. I think loss of independence is probably the biggest [challenge]. I suppose having to rely on others to do a lot of things. Pa22 (aged 43, female, grade 2 astrocytoma)
*Communal specification* People working together to build a shared understanding of the aims, objectives, and expected benefits of a set of practices.	HCP: who takes responsibility (not one individual's responsibility) HCP and people with LGG: perceived importance of support network	Helping [the support recipient] to understand that they have responsibilities too. They can have the responsibility to self‐manage themselves by changing the way that they think. HCP21 (Physiotherapist) We can give information and be a sounding board and we can help them to make a decision, but we can't tell them what the right thing is for them because they know themselves best. We quite often use those phrases, ‘You're the experts about yourself. You know yourself best. So, we can give you the information to help you make a decision but it's your decision’. HCP43 (Specialist allied health professional) There is a large degree of responsibility that falls on family and relatives to keep [the support] going. HCP36 (Occupational Therapist) When you're married and working, you've got your support group and you've got your life. But when you're on your own and quite poorly, you've got nothing. Pa20 (aged 47, female, grade 3 oligodendroglioma)
*Individual specification* The individual's understanding of their specific tasks and responsibilities around a set of practices.	HCP: perceived role in self‐management support (empowering people with brain tumours and family, providing tools, listening to needs to develop goals) **BUT** people with LGG: support is treatment focused	Our role is helping to just make sure the patients have got the information that they need, that they are signposted to information and support about health and wellbeing and helping to make sure that the patients have got good quality of life really. (HCP45, Clinical Nurse Specialist) There's lots of other ways that we can help people to have psychological wellbeing which doesn't necessarily mean a one‐to‐one session, there are leaflets, websites, self‐help, group work, peer support, charity, third sectors, all of that I think is relevant. So, I think I see it as structured or supported self‐management. HCP18 (Clinical Neuropsychologist) It's very much listening and knowing the patient, knowing where they're at in terms of their journey, their pathway and unpicking what sort of things can be put in. So, it's not just a psychological thing, physical thing, it's all of them basically. HCP39 (Clinical Nurse Specialist) There are some things I can do for you. And there's some things you can do for yourself. And what is missing is the second bit from consultations. It's all about what they can do to you to treat your tumour, not treat you as a person. Pa17 (aged 51, female, grade 3 oligodendroglioma)
*Internalisation* Understanding the value, benefits and importance of a set of practices.	HCP: perception of self‐management	Self‐management, as I understand it, is being able to upscale or educate the patient in ways that they can actually look after themselves with regards to any issues that have come in, being aware of red flags that then they would need to contact us. HCP49 (Occupational Therapist) Self‐management to me is about empowering somebody with the right information and resource, access to resource to be able to take more ownership on your health and wellbeing and actually you're saying what you want done basically. HCP39 (Clinical Nurse Specialist) For epilepsy, I would say [self‐management] is more about keeping yourself seizure free so it's about good compliance, keeping yourself healthy and sharing that you've reduced all your triggers and reaching for help as soon as you see a decrease in your seizure control. HCP52 (Epilepsy Nurse Specialist)
*Cognitive participation—The relational work that people do to build and sustain a community of practice*		
*Initiation* Whether or not key participants are working to drive a new set of practices forward.	HCP and People with LGG: engagement in help‐seeking (insight, desire not to be a burden) HCP and People with LGG: signposting to available support People with LGG: support has to be sought	It's hard, isn't it, to say in an appointment, ‘Well, this has really traumatised me. I need some help with this’. It's quite difficult to say that. Pa10 (aged 37, female, grade 2 oligodendroglioma) Patients don't tend to recognise how they've changed, or they don't really want to tell you that something's changed in their needs. HCP48 (Neurooncology Support Sister) I'm actually in the process of developing a leaflet to give to all patients with [support] details on so that they've got it so if and when they feel like they need some support there's options, websites and things. HCP18 (Clinical Neuropsychologist) The lack of information is probably worse—you know, not being able to find it, from not being told about what is available, and not knowing about [hospital] or the outreach services, community outreach, things like that. All of this stuff I have found, basically, it came up by accident. Pa13 (aged 52, male, grade 3 oligodendroglioma) I think it's sometimes like it's for me to put the pieces together. I'm not sure everybody would really be in the position to do that, so I think that's one thing I've learnt about the health service, it's in compartments and joining those compartments is down to you sometimes. Pa28 (aged 66, male, grade 2 astrocytoma)
*Enrolment* Need for people to organise or reorganise themselves and others to collectively contribute to the new practices.	HCP and People with LGG: Influence of support network on self‐management People with LGG: need for reliance on others HCP: availability of/challenges accessing available support	Everybody's got a different story, a different amount of support mechanisms, so some people need more support than others. Some have huge family support and friend support and don't maybe need as much help, really. HCP21 (Physiotherapist) They're only 15 minutes or something when you see your doctor. I'll come out and I won't remember any of it. I always have to have either my dad or my partner with me, or I need it to be written down and summarised or emailed. Pa26 (aged 37, female, grade 2 oligodendroglioma) I only go to the pub, for example, if I'm going with a friend, I could not go on my own to somewhere like that. When I've been on my own and I've had seizures before in the public, well it's something that I really don't like. Pa25 (aged 45, male, grade 2 oligodendroglioma) [People with brain tumours] lack a lot of the support stuff that other cancers get. You know, if you look at breast cancer—the media interest in that, the number of celebrities that have been … you know, brain tumours are the poor relation and have been for years. HCP29 (Consultant Clinical Oncologist) In the community, there are a variety of brain injury services. So, they offer rehabilitation for clients who have neurological conditions. Unfortunately, for many of our clients, they are excluded from those services though, basically because they're considered to have a condition that is progressive. HCP37 (Clinical Neuropsychologist)
*Legitimation* Ensuring that participants believe it is right for them to be involved and they can make a valid contribution.	HCP and People with LGG: resistance to support (beliefs: acceptance, desire for ‘normality’) HCP and People with LGG: support accessibility (location)	Unless people can accept or reach a point where they're kind of accepting they can't really put in those changes or they struggle to really put those changes in and the ones that do better with me are the ones that are working through that grieving process and that process of my identity's completely changing and coming to that place of acceptance. Whereas the ones that I find really hard to engage are the ones that are really struggling with that process. HCP3 (Occupational Therapist) If I'm totally frank there are still lots of moments where I don't really believe [I have a brain tumour], I obviously do believe it, I've had the surgery but where you, I don't know how to describe it, where you look at yourself in the mirror and you think, ‘I can't believe this is happening’. Pa40 (aged 31, female, grade 2 astrocytoma) Each area has different services, so we've still got that postcode lottery problem. HCP43 (Specialist allied health professional) Sometimes brain care teams only support based on the postcode that your doctor is in. This lady came back to me and said, ‘I can't get the support. The doctor is in a different area’. Pa18 (aged 55, female, grade 3 oligodendroglioma)
*Activation* Participants need to collectively define the actions and procedures needed to sustain a practice and stay involved.	HCP: identifying those with support needs (keeping tabs) HCP and people with LGG: opportunity to report needs; need for sustained support HCP and People with LGG: maintaining awareness of available support HCP and people with LGG: resistance to support (actions: nonattendance/compliance)	Once [the support recipient] is in the system and they're known to us, there's options but I guess I'm a bit worried about those whose needs never get identified. HCP18 (Clinical Neuropsychologist) I feel like some patients probably do get lost into the system a little bit and we are reliant on them ringing us and not everybody does that. They just don't want to bother you, or they feel like you might be too busy. HCP21 (Physiotherapist) We introduced, quite a while ago, the screening tool, which has been sent out to compliment that consultation but allows them an opportunity, they should get it before the consultation, so it brings to mind what—so that they think about what difficulties am I having? HCP37 (Clinical Neuropsychologist) I think it depends a lot on individuals. How can they find their way through all this themselves because I think you are, at the moment, thrown back on your own resource system to make your way through this minefield. Pa28 (aged 66, male, grade 2 astrocytoma) I think from our point of view it's probably the information side and probably reiterating where they can get information from later down the line if they don't want to use it there and then because quite often people aren't ready to accept their diagnosis so it's about recapping it later on and catching back up with them. HCP52 (Epilepsy Nurse Specialist) I think the difficulty we have as health professionals is knowing exactly what [support] is out there and what changes and what's available. We do try and have a database, but it changes. So, keeping up to date with what's available as well is quite difficult. HCP21 (Physiotherapist) It's the lack of access to stuff that is probably the biggest problem, and it doesn't help. Not knowing about what you can get access to is probably the biggest problem. Pa13 (aged 52, male, grade 3 oligodendroglioma) You've got your patients who you just try and help, and you try and encourage, but they'll ring you up and they want help, but then they don't accept your advice or offers to signpost or whatever or refer to whatever. HCP33 (Clinical Nurse Specialist)
*Collective action—The operational work that people do to enact a set of practices*		
*Interactional workability* The interactional work that people do with each other and elements of a set of practices, when they seek to operationalise in everyday settings.	HCP: co‐ordination between HCPs (referral pathways) HCP: co‐operation of other HCPs (jointly delivered support) People with LGG: communication between HCPs HCP and People with LGG: including family in support provision	The nurses will sometimes refer people in. Most of our referrals come from the inpatient occupational therapists. We really struggle with getting the consultants to refer to us. I don't know why. HCP3 (Occupational Therapist) Everything's being dealt with as a separate entity. And I think if we can get one service to merge into another, to then merge into another, that line of communication, that line of smoothness, I think we'd be looking at increasing somebody's quality of care with these brain tumours. HCP1 (Clinical Nurse Specialist) [neuro‐consultant] dealt with the first bit of [care] and then she handed me on to, they had lots of multidisciplinary team meetings and you're part of that, and then you get passed onto the appropriate person, in this case it was the neurosurgeon. And so, information seemed to get lost in some of that. You're sort of passed around. Pa5 (aged 56, male, grade 2 oligodendroglioma) Where possible we try and involve family in discharge planning discussions and kind of future planning discussions. We involve them as much as we can because it's as much their tumour as it is the patient's tumour in some senses, especially when you're looking at the low‐grade tumours that have got quite a long life expectancy. HCP36 (Occupational Therapist) [partners] need to understand what's going on. They need to be supporting you, yes, definitely. I don't know where I'd be without my wife. Pa33 (aged 45, male, grade 2 oligodendroglioma)
*Relational integration* The knowledge work that people do to build accountability and maintain confidence in a set of practices and in each other as they use them.	HCP and people with LGG: importance of HCP‐support recipient relationships (social skills, trust, reassurance)	Some of my patients, they feel as if you're the one … because I do my job, I'm the one who should be there for their beck and call. I'm the one that should be making the decisions. I'm the one that, you know, that's my job. They don't particularly take on responsibility. HCP2 (Clinical Nurse Specialist) We think that you're at this level and this is why and just giving them examples. But you have to have a good rapport with that patient to be able to talk like that. It's a fine line between agitating them and trying to get them to realise that there's a problem. HCP44 (Occupational Therapist) I'm sure that all [healthcare professionals] are medically highly skilled, but obviously that's not the only bit of the job is it. You have to understand people. Understand how they're're feeling. Know how to speak to them. Make them feel reassured at what is a very frightening period in their life. Pa28 (aged 66, male, grade 2 astrocytoma) As soon as we went in there [the consultant] was almost like, ‘I want to put your mind at rest about this’, kind of thing. Even though it's become more serious … I think she even said, ‘The treatment for this we can get for you is better’. So, it's worse but we can do more for you for it, kind of thing. Whatever she said was really reassuring. Pa32 (aged 46, female, grade 3 oligodendroglioma)
*Skill set workability* The allocation work that underpins the division of labour that is built up around a set of practices as they are operationalised in the real world.	HCP: expertise and training needs HCP and people with LGG: cognitive and symptom challenges HCP and People with LGG: health and technology literacy	If you are managing or leading a service, you sometimes lead to your strengths, just as I am doing. And our support, unknowingly, has been missing out on a few key aspects because that's not within my expertise. HCP17 (Macmillan centre manager) It's very hard to self‐manage if a memory deficit is there because at the end of the day a prompt is needed to set the prompt. Because she couldn't write the list herself or couldn't set the phone reminders reliably. So, I think if you're truly isolated, that has a massive impact on how successful you are going to be. HCP28 (Consultant Clinical Oncologist) The reminders on the iPhone are good but you try to remember things. That again that adds to your brain ache if you like, you're trying to collate all this stuff while you're worrying about it. Pa5 (aged 56, male, grade 2 oligodendroglioma) My concern is those people that need more than [support groups] or can't access it for barriers of communication, digital, financial or cognitive or visual or purely because they don't have the digital knowledge to be able or the wish to access the support in that way. HCP50 (Specialist allied health professional) All sorts of ages and technical competences and some people won't [access the support], if they've got to do it online. It's hard, it's difficult. Pa16 (aged 69, male, grade 3 oligodendroglioma)
*Contextual integration* Managing a set of practices through the allocation of different kinds of resources and the execution of protocols, policies, and procedures.	HCP: financial and equipment resources HCP: time, flexibility, and waiting lists HCP: staff availability People with LGG: transport and financial resources	Financial I suppose is just about the biggest and probably the only [challenge], you could say this for any service across the NHS, couldn't you, that it could be better and more comprehensive in an ideal world if there were more resources. HCP51 (Speech and Language Therapist) There is always room for service improvement. There is always room to benchmark against other centres, and to improve outcomes for patients, and I think it's just … sometimes that gets lost in amongst the rotation of just dealing with everyday … day to day things, you know. HCP14 (Clinical Nurse Specialist) Our low‐grade glioma patients, quite often after their surgery, the only person that's in contact with them is me or the physio. The support pathway after that isn't very well established or there. Then after me, they might have a three‐month post‐op meeting with the consultant but that will be the next time they see someone. HCP3 (Occupational Therapist) When I saw my naturopath … and again, I was paying, like, £60 a session. So, I saw him for a while, but I couldn't … and then in between there was always things to buy and it just got too expensive. Pa17 (aged 51, female, grade 3 oligodendroglioma)
*Reflexive monitoring—The appraisal work that people do to assess and understand the ways that a new set of practices affect them and others around them*		
*Systemisation* Participants seek to determine how effective and useful the set of practices are for them and for others.	HCP and people with LGG: need to avoid information overload HCP: developing support recipient identity and control HCP: managing expectations	They do say that [the amount of information] is too much. You know, quite often, we … well, I'll acknowledge, and they will acknowledge that I have given them a huge amount of information. HCP29 (Consultant Clinical Oncologist) In an ideal world you'd have all of this information at your fingertips because anybody with a brain tumour doesn't want to receive a plethora of post with loads of paper because you're still getting to grips with the fact that you have a debilitating, longstanding illness and it's a tumour and it's cancerous. Pa18 (aged 55 female, grade 3 oligodendroglioma) So that they can take control and they are part of the decision‐making, rather than just a person or a number. It's bringing it to life, isn't it, and empowering them to see that they can take an active participation in that treatment and support. HCP17 (Macmillan centre manager) One of the lessons that I need to learn and to remind myself of, is the importance of self‐care which can be done in a way that is not selfish in orientation but needful to make the most of. You can't give to the world if you're not giving to yourself in a way. Pa14 (aged 66, male, grade 2 oligodendroglioma) With a lot of the brain tumour patients we've got to help them to be quite realistic and support that as an ongoing thing. So, I guess it's important to address that although support might help them, it's not going to fix all of their problems but help them manage their problems themselves. HCP36 (Occupational Therapist)
*Communal appraisal* Participants work together to evaluate the worth of a set of practices.	HCP: scope of support provision (number reached; variation in needs) HCP: clarify benefits to end‐users	The numbers are low. So, I get some rare cancer types and people go, ‘I just want to talk to another person with the same cancer as me’. That person is probably in Scotland, or that person is in Brighton. You know, the numbers just aren't there. HCP17 (Macmillan centre manager) [Offered support] is going to depend on the individual, where they're at in their journey and what they need and what they prefer as well. HCP18 (Clinical Neuropsychologist) I think self‐management would need to be promoted as a really … ‘This is the reason we are doing it. It's a really positive thing, and we need you to help us identify these bits, because we can positively make a difference. We can help you by x, y, and z’. So, in a way, it's about telling people why you are doing it—‘with this in mind … it's going to make a difference because…’ HCP29 (Consultant Clinical Oncologist)
*Individual appraisal* Participants work experientially as individuals to appraise its effects on them and the contexts in which they are set.	HCP and people with LGG: perception of support groups (social comparisons) People with LGG: appreciation of self‐management support	The Maggie's centre works with the cancer nurse specialists, and they have some great [support groups], so we encourage and suggest those groups or look at where the patient lives. HCP17 (Macmillan centre manager) Some people if they've got a low‐grade tumour and they're aware that could progress over time, might not want to face that in a support group really. They might want to deal with it themselves. So, they might not want to see people further down the line and sort of see what's going to happen. HCP45 (Clinical Nurse Specialist) The thing with brain tumour sufferers, patients or whatever, is that everyone's different. Everyone, because the brain is such a complicated organ, depending on where it is, what it is, everyone's different. It's so difficult to get common ground with other brain tumour patients. Pa5 (aged 56, male, grade 2 oligodendroglioma) I go on the brain tumour support groups. So, I use that for a lot of information. Plus, you can give advice to other people and people can give advice to you of their experiences and that which is quite a good help sometimes. Pa30 (aged 61, male, grade 3 oligodendroglioma) The thing is I know that if that situation changes, then the good thing is through the medical team, the multi‐district team at [hospital], through family and friends, and through things like The Brain Tumour Charity, that there will be the support for me, should those situations change. Pa15 (aged 55, male, grade 2 astrocytoma)
*Reconfiguration* Appraisal may lead to attempts to redefine procedures or modify practices.	HCP: including family requires careful consideration (need permission)	We'll quite happily, if someone wants us to speak to their partner, as long as [the support recipient] gives permission we'll give them a ring and chat through. HCP52 (Epilepsy Nurse Specialist) I've had an issue where I had a lady and a son and daughter, she couldn't communicate together. And basically, the patient was, like, piggy in the middle. And I would have the three of them phoning every single day, even discovering different versions of the story, and it became very intense for myself, and I tried to offer advice that only one of them liaise with mum and it's about working together as a family. HCP48 (Neurooncology Support Sister) Occasionally, we get ex‐partners, and we don't know whether the patient wants us to talk to them. It can come from a bad place. So, we do try to be careful to check that the patient is okay with us talking. HCP28 (Consultant Clinical Oncologist)

Abbreviations: HCP, healthcare professional; LGG, lower‐grade glioma.

As NPT is a theory more traditionally used to describe and explain implementation as the activity of professional providers, we started by mapping the HCP data to the NPT constructs, then interrogated each of these mappings with reference to the experiences and perspectives of the support recipients. We have explored and reported elsewhere the self‐management strategies used by people with LGG.^13^ Here, we wanted to explicitly explore the alignment (or otherwise) of the data from people with LGG with HCPs' experiences and perspectives relating to issues of SMS implementation. If data relevant to a particular (sub‐)construct was seen amongst people with LGG, but had not been raised by HCPs, this was added to the analysis.

## 3 RESULTS

### 3.1 Participant characteristics

Fifty‐two HCPs registered interest in taking part, 46 were eligible (not involved in the support of adults with brain tumours, *n* = 4; and practised outside the United Kingdom, *n* = 2), and 25 were purposively selected for interview (recruitment route: NHS sites *n* = 16; The Brain Tumour Charity *n* = 9). During the interview, participants had on average 11.6 years (range 1–25 years) experience working with people with brain tumours. Participants worked across nine geographical regions of the United Kingdom and 12 different healthcare professions, most commonly: Clinical Nurse Specialist (*n* = 6), Occupational Therapist (*n* = 4) and Clinical Neuropsychologist (*n* = 3) (Table 2).

**Table 2:** Healthcare professional participants' characteristics at the time of the interview.

Characteristic	*n*	Characteristic	*n*
*Profession*		*Geographical region*	
Clinical Nurse Specialist	6	Tyne and Wear	9
Occupational Therapist	4	North Yorkshire	4
Clinical Neuropsychologist	3	Lothian	3
Consultant Clinical Oncologist	2	Greater Manchester	3
Physiotherapist	2	South Wales	2
Specialist allied health professional	2	Leicestershire	1
Consultant Neurosurgeon	1	Merseyside	1
Consultant Neuroradiologist	1	Shropshire	1
Epilepsy Nurse Specialist	1	West Yorkshire	1
Macmillan Centre Manager	1		*Mean* (*range*)
Neurooncology Support Sister	1	*Time working with people with brain tumours* (*years*)	11.6 (1–25)
Speech and Language Therapist	1		

Thirty‐nine people with LGG registered interest in taking part, 35 were eligible (noncompletion of primary treatment, *n* = 2; residence outside the United Kingdom, *n* = 1; and ineligible diagnosis, *n* = 1), and 28 were purposively selected for interview (recruitment route: NHS sites *n* = 10; The Brain Tumour Charity, *n* = 18). At interview, participants were aged on average 50.4 years (range 22–69 years); male (*n* = 16), female (*n* = 12); diagnosed with a grade 2 oligodendroglioma (*n* = 10: IDH1‐mutant, yes *n* = 7, no *n* = 2, unknown *n* = 1; 1p/19q codeletion, yes *n* = 9, unknown *n* = 1), grade 3 oligodendroglioma (*n* = 9: IDH1 mutant, yes *n* = 6, no *n* = 1, unknown *n* = 2; 1p/19q codeletion, yes *n* = 7, unknown *n* = 2), or grade 2 astrocytoma (*n* = 9: IDH1‐mutant, yes *n* = 6, no *n* = 1, unknown *n* = 2; 1p/19q codeletion, no *n* = 7, unknown *n* = 2) (Table 3). The average time since diagnosis was 8.7 years (range 1–18 years); the treatment received were surgery (*n* = 28), radiotherapy (*n* = 22) and chemotherapy (*n* = 17).

**Table 3:** Lower‐grade glioma participants' characteristics at the time of the interview.

Characteristic	*n*	Characteristic	Mean (range)
*Diagnosis* a		*Time since diagnosis (years)* a	8.7 (1–18)
Grade 2 oligodendroglioma	10	*Time since radiotherapy (years)* ^a^, ^b^	6.9 (0.7–17.8)
Grade 3 oligodendroglioma	9	*Time since chemotherapy (years)* a,b	3.4 (0.1–13.5)
Grade 2 astrocytoma	9	*Full‐time education (years)*	15.8 (11–20)
*IDH‐mutation status* a		*Sex*	*n*
Yes	19	Female	12
No	4	Male	16
Unknown^c^	5	*Age*	
*1p/19q codeletion status* ^a^, ^d^		≤40	4
Yes	16	41–50	8
No	7	51–60	11
Unknown^c^	5	>60	5
*Treatment* a		*Dependents*	
Surgery	28	None	18
Radiotherapy	22	One	3
Chemotherapy	17	Two	6
*Tumour location* a		Three	1
Frontal	18	*Employment status*	
Temporal	3	Full‐time employee	8
Parietal	3	Part‐time employee	4
Overlapping regions	3	Retired	4
Unknown	1	Medically retired	6
*Tumour laterality* a		Unable to work	6
Right hemisphere	13	*Relationship status*	
Left hemisphere	15	Married	21
Dominant hemisphere	13	In a relationship	3
Nondominant hemisphere	15	Single	2
		Widowed	2

^a^ Clinical and tumour‐related details were self‐reported for eight participants.

^b^ Time since radiotherapy and chemotherapy were not available for two participants.

^c^ Some participants were diagnosed before mutation status was routinely assessed.

^d^ All participants with 1p/19q codeletion were people with oligodendroglioma; all participants without 1p/19q codeletion were people with astrocytoma.

### 3.2 Overview of findings

HCPs spoke about how the impact of the tumour and its treatment on people with LGG (e.g., cognitive deficits) created specific challenges for effective engagement in self‐management for this population. The data we generated mapped extensively to all four NPT constructs and related subconstructs; some subconstructs were more supported by the data than others (Table 1). Our findings, described below by construct with supporting quotes throughout, outline the considerations required to operationalise, sustain and appraise the implementation of SMS for people with LGG.

#### 3.2.1 Coherence

Coherence encompassed HCPs' sense‐making of self‐management, and their perceived role and responsibilities in providing SMS. Most HCPs expressed an internalised perception of the importance of providing SMS; they highlighted the value of ‘empowering’ people with LGG to ‘look after themselves’ beyond the clinical care setting.

Self‐management to me is about empowering somebody with the right information and resource, access to resource to be able to take more ownership on their health and wellbeing, and actually [the support recipient] is saying what they want done basically. HCP39 (Clinical Nurse Specialist)

The key differentiation between self‐management and other healthcare support was HCPs' perception that supporting self‐management is about promoting independence, so that people with LGG can ‘take responsibility’ for managing their condition. However, the desire to promote independence may not always be achievable; several people with LGG described how they experienced challenges with a loss of independence and reported having to be reliant on others (e.g., due to losing their driving licence), which was not always something they desired.

The loss of my licence and independence. I think loss of independence is probably the biggest [challenge]. I suppose having to rely on others to do a lot of things. Pa22 (aged 43, female, grade 2 astrocytoma)

Most HCPs outlined an understanding of the specific role and responsibilities they have in supporting self‐management, working with the person's own realities and preferences to provide personalised support. This included: appropriate signposting to information and support; providing tools (i.e., suggesting specific self‐management strategies) to empower both people with LGG and families; and listening to individual's needs to co‐develop goals.

It's very much listening and knowing the patient, knowing where they're at in terms of their journey, their pathway and unpicking what sort of things can be put in. So, it's not just a psychological thing, physical thing, it's all of them basically. HCP39 (Clinical Nurse Specialist)

However, some people with LGG felt that the support received was focused on ‘what they can do to treat your tumour, not treat you as a person’.

Several HCPs spoke about a desire to build a shared understanding with people with LGG that self‐management is not one individual's responsibility; they acknowledged the importance of a communal approach to self‐management, with people with LGG assuming their own responsibilities, and close family and friends assuming support responsibilities in the home environment. Aligned with this, people with LGG recognised that the role and strength of their support networks influenced their ability to engage with self‐management, for example, through the provision of practical support with housework and transport.

#### 3.2.2 Cognitive participation

Cognitive participation encompassed the relational work of HCPs and people with LGG to build and sustain a ‘community’ of supporting self‐management. To initiate SMS, both HCPs and people with LGG outlined the importance of signposting to relevant information and available support. However, many people with LGG expressed that this was often lacking and that they had to proactively seek support. Several HCPs conveyed that, due to a lack of insight (often because of the impairments that the tumour can cause) or desire not to be a burden, some people with LGG do not seek the help they need. People with LGG contended that help‐seeking can be difficult to initiate within the opportunities presented.

Patients don't tend to recognise how they've changed, or they don't really want to tell you that something's changed in their needs. HCP48 (Neurooncology Support Sister)

It's hard, isn't it, to say in an appointment, ‘Well, this has really traumatised me. I need some help with this.’ It's quite difficult to say that. Pa10 (aged 37, female, grade 2 oligodendroglioma)

People with LGGs' initiation of self‐management, and HCPs' perception of the amount of support required, was also influenced by the presence and strength of the support recipient's support network to collectively contribute to their self‐management. For example, one person with LGG felt unable to go out in public without company, in case they had a seizure.

Several HCPs stated that the support available to which people with LGG could be signposted is poor in comparison to other cancers; where brain injury rehabilitation services were available, people with LGG were often excluded due to the progressive nature of their condition. HCPs and people with LGG similarly reported that access to support can also be influenced by the services available within the person's location.

Each area has different services, so we've still got that postcode lottery problem. HCP43 (Specialist allied health professional)

HCPs spoke about how their perception of whether they could make a valid contribution to supporting self‐management was influenced by challenges with the support recipient's acceptance. This was corroborated by some people with LGG who described how they were struggling to process the consequences of their condition and were resistant to having an active role in their own self‐management.

To collectively sustain engagement in (supporting) self‐management, HCPs and people with LGG acknowledged the importance of regular opportunities to report support needs to someone involved in their care (e.g., through a screening tool) and having ways to maintain awareness of available support beyond initial signposting. Several HCPs outlined difficulties with ‘keeping up to date’ with what support is available, especially in the community (e.g., charities); changes in available support evoked challenges with distributing information resources.

I think the difficulty we have as health professionals is knowing exactly what [support] is out there and what changes and what's available. We do try and have a database, but it changes. So, keeping up to date with what's available as well is quite difficult. HCP21 (Physiotherapist)

Most HCPs expressed concerns with keeping track of support needs, particularly of those that do not seek help, stating the importance of active participation from both HCP and people with LGG to recognise needs and sustain SMS. Still, some HCPs outlined that there are people who seek support, but then resist the support that is offered.

You've got your patients who you just try and help, and you try and encourage, but they'll ring you up and they want help, but then they don't accept your advice or offers to signpost or whatever or refer to whatever. HCP33 (Clinical Nurse Specialist)

#### 3.2.3 Collective action

Collective action encompassed the considerations required to operationalise the implementation of SMS, to ‘make it work’ in practice. Several HCPs highlighted the importance of coordination between HCPs for them to become aware of someone with support needs; this was particularly important for allied HCPs offering a specific service (e.g., occupational therapy). People with LGG expressed that communication between HCPs was often not streamlined, with information getting lost as they were ‘passed around’. Some HCPs echoed this sentiment and suggested that quality of care would improve with improved HCP cooperation, rather than dealing with each support need as a ‘separate entity’.

Most HCPs expressed the value of building a good rapport with their support recipients to facilitate effective communication about their support needs. People with LGG appreciated when HCPs showed strong social skills; this generated trust and helped them feel reassured.

I'm sure that all [healthcare professionals] are medically highly skilled, but obviously that's not the only bit of the job is it. You have to understand people. Understand how they're feeling. Know how to speak to them. Make them feel reassured at what is a very frightening period in their life. Pa28 (aged 66, male, grade 2 astrocytoma)

Still, some HCPs spoke about how there is a ‘fine line’ in these relationships; some HCPs wanted people with LGG to assume more responsibility in the decision making concerning desired support.

Both HCPs and people with LGG outlined the value of including close family and friends in support to ensure they understood the condition and its consequences, and how they can be involved in supporting self‐management. Particularly, both groups identified challenges with cognitive deficits that mean additional assistance in the home environment can be beneficial.

It's very hard to self‐manage if a memory deficit is there because at the end of the day a prompt is needed to set the prompt. Because she couldn't write the list herself or couldn't set the phone reminders reliably. So, I think if you're truly isolated, that has a massive impact on how successful you are going to be. HCP28 (Consultant Clinical Oncologist)

Both HCPs and people with LGG outlined the implications of poor technology literacy on access to, and engagement with, support, acknowledging that ‘some people won't [access the support] if they've got to do it online’. Several HCPs identified their own training needs to deliver support, linking this to the need for cooperation between HCPs; they noted that if they were not trained to provide particular types of support, or not aware that support could be provided for particular problems beyond their expertise, this would often be missing from their service.

If you are managing or leading a service, you sometimes lead to your strengths, just as I am doing. And our support, unknowingly, has been missing out on a few key aspects because that's not within my expertise. HCP17 (Macmillan centre manager)

All HCPs stressed the impact of resources on the execution of support provision. This largely encompassed financial constraints within the service, and time; the lack of availability and flexibility of HCPs meant several HCPs felt unable to ‘benchmark against other centres’ to improve their services or maintain the desired continuity of care. Most people with LGG expressed additional concerns, primarily with transport challenges to attend support, and their own financial resources to acquire equipment.

#### 3.2.4 Reflexive monitoring

Reflexive monitoring encompassed HCPs' appraisal of the worth and effectiveness of implementing SMS. Several HCPs acknowledged the need to avoid information overload when providing SMS, as sharing too much information at once could be overwhelming for the recipient. Similarly, people with LGG reflected on the importance of appropriately timed information sharing, and the need to consider the time it takes to accept their condition; they suggested it would be most effective to have access ‘at your fingertips’ for when it is required.

HCPs determined that implementing SMS was useful for empowering people with LGG to take an active role in their care. An important element of this was managing the expectations of people with LGG to help them work towards realistic goals.

With a lot of the brain tumour patients we've got to help them to be quite realistic and support that as an ongoing thing. So, I guess it's important to address that although support might help them, it's not going to fix all of their problems but help them manage their problems themselves. HCP36 (Occupational Therapist)

Most HCPs outlined how the relative rarity of LGGs, and the wide‐ranging support needs of people living with these tumours present challenges for the types of SMS that can be provided; for example, opportunities for people with LGG to share advice and experiences with similar others may be hindered by their disparate locations. Where group support was available for people with LGG, attendance was tentatively encouraged by HCPs, with the awareness that individual journeys and preferences may influence the value of this type of support; this was corroborated by people with LGG:

Some people if they've got a low‐grade tumour and they're aware that could progress over time, might not want to face that in a support group really. They might want to deal with it themselves. So, they might not want to see people further down the line and sort of see what's going to happen. HCP45 (Clinical Nurse Specialist)

The thing with brain tumour sufferers, patients or whatever, is that everyone's different. Everyone, because the brain is such a complicated organ, depending on where it is, what it is, everyone's different. It's so difficult to get common ground with other brain tumour patients. Pa5 (aged 56, male, grade 2 oligodendroglioma)

People with LGG expressed considerable appreciation for available support, once they were aware of potential avenues of access; for some, the knowledge that resources were available should they be needed in the future, was highly valued. Several HCPs acknowledged a need to promote self‐management and explain the potential benefits to people with LGG to improve understanding of why self‐management is important, and how HCPs and people with LGG can work collectively to make a difference. HCPs highlighted that including close family and friends into the collective action of SMS requires careful consideration; family often assumed the responsibility of help‐seeking without permission from the support recipient, leading HCPs to reconfigure the involvement of family.

We'll quite happily, if someone wants us to speak to their partner, as long as [the person with the brain tumour] gives permission we'll give them a ring and chat through. HCP52 (Epilepsy nurse specialist)

## 4 DISCUSSION

People with LGG can have complex, multi‐faceted supportive care needs.^10^ Amongst people with cancer, clinical and psychosocial outcomes can be improved through effective engagement in self‐management^29^; this requires support from HCPs, family, and friends.^4^ The considerations required to implement SMS for people with LGG are poorly understood. We aimed to identify and understand these implementation considerations, through the lens of NPT, from the perspectives of HCPs and people with LGG.

We generated extensive supporting evidence for all four NPT constructs and related subconstructs, namely: ‘Coherence’, ‘Cognitive participation’, ‘Collective action’ and ‘Reflexive monitoring’. Overall, our findings demonstrate the ways that SMS for people with LGG should be understood as a collective and collaborative activity. We offer important insights into: (1) how people with LGG can both be supported and enabled to support themselves effectively through service provision; and (2) the challenges that need to be addressed to facilitate implementation of SMS.

In our findings, HCPs recognised the value of their role in self‐management. This included providing the information and support to promote independence and empower people to confidently engage in self‐management. Integrating the perspective of people with LGG highlighted that independence can be difficult to maintain or achieve. This, in turn, emphasises a need for sustained support over time. There are potential organisational constraints with HCP time and flexibility in relation to being able to assess individual's support needs, and maintain SMS provision, over the longer‐term. HCPs emphasised that participation goes both ways, requiring help seeking from the support recipient, so that HCPs can identify and maintain awareness of supportive care needs. This may be influenced by the possibility that people with LGG could underestimate, and thus not seek help for, cognitive, psychological or social changes.^30^ Still, people with LGG outlined the importance of building trust in their relationships with HCPs and expressed difficulties with reporting needs within the opportunities provided, as these are often focused on the treatment for the tumour. Particularly, psychological support is a challenging aspect of SMS to implement and embed for support providers, as this is an area that people reported to be especially difficult to seek and access help for.

Our findings also highlight the critical importance of interaction between HCPs to operationalise SMS provision. HCPs and people with LGG recognised key areas for improvement in the structure and provision of services, including: improved referral pathways to allied HCPs (e.g., Occupational Therapists); encouraging (and enabling) cooperation between HCPs to deliver combined support, where possible and communication between HCPs, so that there is interdisciplinary awareness of what support is needed by, and what has already been provided for, the support recipient. Altogether, these considerations would help ensure SMS is more integrated and holistic enabling people to be more effectively supported to self‐manage. Further, our findings also acknowledge that HCPs may have training needs, and support provision may be influenced by locally available expertise; hence, consideration of a SMS training programme could be beneficial. Such a programme has improved confidence in SMS provision for HCPs in other cancers.^31^ In the United Kingdom, the Tessa Jowell Academy Programme connects brain tumour centres and provides a space to tackle challenges^32^; this is an example of a platform that could help support emergent training needs.

A pertinent challenge to the continuous provision of SMS was the availability of support services for people with LGG; from both perspectives, this was influenced by location, eligibility and keeping up to date with the changing landscape of available support. Where support was available and signposted, HCPs highlighted difficulties with the willingness of people with LGG to engage with the support being offered. Both HCPs and people with LGG recognised that acceptance of the diagnosis influenced engagement with SMS^33^; this indicates the importance of appropriately timed information sharing, so that people can access support when they are ready.^34^ However, a 2021 environmental scan found that most online self‐management resources concern active treatment, with few specifically directed at people with brain tumours, particularly those with LGG, living longer‐term.^35^ Our findings also outlined further challenges with accessibility, due to technology literacy and cognitive deficits; hence, online support may be lacking, not preferred, or require support to access.^36^

We outline, from both perspectives, the pivotal role of a person's support network in bridging the gap between provision of SMS from the HCP, to the implementation of self‐management strategies in the home environment; this was particularly important for those with cognitive deficits. Thus, available support may be a key determinant of successful self‐management for people with LGG. In further analyses from the Ways Ahead project, to be reported elsewhere, we have found from interviews with informal caregivers that family and friends provide wide‐ranging support (e.g., practical, emotional, cognitive) for people with LGG. Nonetheless, the data reported in the current paper suggests that including informal caregivers in SMS requires careful consideration to ensure self‐management remains person‐centred. Informal caregivers may have their own perspectives of what is, or should be, important to the support recipient, and these should not dominate the priorities of the people with LGG themselves. Moreover, informal caregivers may have their own supportive care needs, particularly related to emotional burden,^37^, ^38^ and, while they are important, how these can best be met requires further consideration. For example, informal caregivers of people with a brain tumour desire stronger connections with HCPs to help them feel able to provide support.^39^ While we acknowledged the value of this in our findings, we would concur with HCPs in our study who stressed the importance of permission from the support recipient to ensure inclusion of informal caregivers into SMS is appropriately managed.

Overall, we have an advanced understanding of the mechanisms of SMS implementation by demonstrating and emphasising the collective nature of SMS for people with LGG. Due to the importance of engagement from HCPs, people with LGG, and informal caregivers, it is crucial that SMS is seen as a collective activity, requiring the kinds of interactions and communications that support effective self‐management for people with LGG. Improved partnership working between HCPs and people with LGG also needs to recognise the importance of the autonomy, agency, and capacity of the support recipient. This closer partnership might be facilitated by providing HCPs with the skills and confidence to work with peoples' concerns that are ‘non‐treatment focused’. Co‐created with service users to develop more personalised models of care, the Bridges approach to SMS emphasises the collaborative nature of SMS and involves training practitioners to use language and other techniques as part of everyday practice.^40^ In stroke and neurorehabilitation across 24 UK NHS Trusts, successful implementation and integration of the Bridges approach to supported self‐management demonstrated increased skills and confidence in providing SMS. Still, a key distinction between care pathways for stroke patients and people with LGG is the incurable nature and likelihood of progression in people with LGG, which may influence the approach to rehabilitation.

Viewed as collective activity, SMS must be acceptable and feasible for all stakeholders. This underlines the need for a detailed understanding of the desired support and design preferences for SMS from the perspective of each stakeholder. For example, support groups may appear to be a valuable platform for sharing advice and experiences; however, functional challenges (e.g. location, timing) and issues with heterogeneity across people with LGG, may preclude engagement, which has potential implications for the scope of providing group support. Ultimately, future development of supported self‐management interventions for people with LGG^26^ should include comprehensive co‐design with all stakeholders, to acknowledge and look to overcome the challenges and constraints highlighted in our findings. In other research guided by NPT, Mäkelä et al.^41^ demonstrated the feasibility of co‐designing SMS approaches with people with traumatic brain injury (TBI) and showed promise in addressing implementation challenges related to complex service pathways for people with TBI.

### 4.1 Strengths and limitations

The overarching strength of our study was the novel contribution to a very limited evidence base concerning the implementation of SMS for people with LGG. This was underpinned by several methodological strengths, including: (1) our application of NPT, which provided a deeper understanding of the mechanisms of social action, that underpin implementation processes^42^; (2) involvement of multiple stakeholders (HCPs and people with LGG), which allowed us to understand SMS implementation considerations from the perspectives of service providers and support recipients^43^; (3) inclusion of a wide range of healthcare professions, which helped us capture the challenges faced by different roles within SMS provision and (4) inclusion of HCPs from numerous regions across the United Kingdom, which provided diverse experiences with implementation challenges from within different provider settings, representing different levels of organisation readiness to support self‐management.

A key limitation of our study, however, was that people with LGG with more limited capacity may have been discouraged from taking part, due to the expected interview length (approximately 90 min). This means those with greater support needs or challenges engaging with self‐management, may have been missed; multiple, shorter interviews could be considered in future research to mitigate risk of fatigue. Moreover, although telephone interviews have previously been shown to be effective for discussing sensitive issues,^44^ in this context, in‐person interviews may have helped the interviewer to better gauge the impairment of the participant.

Our eligibility criteria included people with grade 3 oligodendrogliomas; while such diagnoses would not necessarily be considered low‐grade, we included them because they have a similar prognosis to those with low‐grade brain tumours.^8^ The people with LGG were up to 18 years postdiagnosis. Molecular assessment of tumours became routine after some of the participants were diagnosed, and for that reason, we included participants with either eligible molecular features or a diagnosis based on histology only. Recruitment across the United Kingdom and through The Brain Tumour Charity networks means that HCPs and people with LGG involved in this study could be in different services; hence, some instances where people with LGG provided contrasting experiences to the practice described by HCPs might be because their local services did not include the elements, or did not operate in the way, described by the interviewed HCPs. Further, HCPs often spoke more widely about the services provided for people with brain tumours rather than focusing only on LGG. Those with different types of brain tumours may share similar experiences of impairments, functional limitations, and reduced life expectancy.^45^ Therefore, our findings are likely to also have relevance for SMS provision for people with other types of brain tumours.

## 5 CONCLUSION

This study provided, for the first time, a comprehensive insight into the collective nature of, and individual roles within, supported self‐management and outlined the considerations required to operationalise, sustain and appraise the implementation of SMS for people with LGG, through the lens of NPT. This provides a crucial first step towards creating a shift in care culture to embed co‐created SMS, by shedding light on factors influencing implementation that need to be overcome at the organisation, HCP and support recipient levels. Our findings can inform the development of supported self‐management interventions for people with LGG, ensuring these have a line of sight to future implementation into routine care.

## AUTHOR CONTRIBUTIONS

**Ben Rimmer**: Writing—original draft; investigation; methodology; writing—review and editing; project administration; formal analysis. **Tracy Finch**: Conceptualisation; funding acquisition; writing—review and editing; formal analysis; supervision. **Michelle Balla**: Formal analysis; methodology; writing—review and editing. **Lizzie Dutton**: Methodology; investigation; writing—review and editing. **Sophie Williams**: Conceptualisation; funding acquisition; writing—review and editing; investigation. **Joanne Lewis**: Conceptualisation; funding acquisition; writing—review and editing; investigation. **Pamela Gallagher**: Conceptualisation; funding acquisition; writing—review and editing. **Richéal Burns**: Conceptualisation; funding acquisition; writing—review and editing. **Vera Araújo‐Soares**: Conceptualisation; funding acquisition; writing—review and editing. **Fiona Menger**: Writing— review and editing; supervision. **Linda Sharp**: Conceptualisation; supervision; writing—review and editing; funding acquisition; formal analysis; project administration.

## CONFLICT OF INTEREST STATEMENT

The authors declare no conflict of interest.

## ETHICS STATEMENT

The study was approved by the Wales Research Ethics Committee (REC ref: 20/WA/0118). Informed consent was obtained from all individual participants included in the study. All participants signed informed consent regarding publishing their anonymised data.

## Supporting information

Supporting information.

Supporting information.

## Data Availability

The data that support the findings of this study may be available from the Chief Investigator (Professor Linda Sharp; linda.sharp@ncl.ac.uk) upon reasonable request.
