# Supplementary material for: Understanding supported self‐management for people living with a lower‐grade glioma: Implementation considerations through the lens of normalisation process theory
Source: Health Expect. 2024 May 11;27(3):e14073. doi: 10.1111/hex.14073 (PMC11087884; doi:10.1111/hex.14073)
Supplement: Supplementary file 1 — Supporting information. [file HEX-27-e14073-s001.pdf]

Topic guide for healthcare professional interviews

The direction and content of the interview, the order in which topics are covered, and the precise wording of questions and probes, will be determined by the issues and topics raised by, and the personal circumstances and experiences of, the interviewee. This topic guide therefore functions as an issue checklist for the interviewer.

Introductory questions:

Would you like to start by telling me a bit about your professional role, and how/when you interact with people with brain tumours?

Topics to cover:

- Issues faced by people living with a brain tumour. Can you illustrate this with particular examples of patients/cases?
- How patients get referred to them
- Available support following treatment completion
    - Patients' unmet needs: physical, emotional, cognitive, social/role, etc.
        - How these needs are identified
    - How patients are made aware of available support
    - Collaboration with other healthcare professionals
    - Barriers to provision of support
    - How patients could be better supported
- Views on self-management for people living with a brain tumour
    - Role of patients, family, health professionals and others in self-management
    - How patients respond to self-management
    - Any supported self-management already provided
- Supported self-management interventions
    - Feelings about referring patients to a self-management intervention
    - Intervention feasibility
        - System/service issues what would hinder implementation
        - What would need to be in place to support implementation
        - What might encourage patients to participate in a self-management intervention

Closing questions:

Is there anything you would like to tell me that we haven't already discussed?
Do you have any questions for me?
